# Evaluation of the Spermatogenic Activity of Polyherbal Formulation in Oligospermic Males

**DOI:** 10.1155/2018/2070895

**Published:** 2018-07-25

**Authors:** Syed Ammar Hussain, Ahsan Hameed, Furqan Nasir, Yang Wu, Hafiz Ansar Rasul Suleria, Yuanda Song

**Affiliations:** ^1^Colin Ratledge Center for Microbial Lipids, School of Agriculture Engineering and Food Science, Shandong University of Technology, Zibo 255049, China; ^2^Mayo Hospital Affiliated with King Edward Medical University, Lahore, Pakistan; ^3^UQ Diamantina Institute, Translational Research Institute, Faculty of Medicine, The University of Queensland, 37 Kent Street Woolloongabba, Brisbane, QLD 4102, Australia; ^4^Department of Food, Nutrition, Dietetics and Health, Kansas State University, Manhattan, KS 66506, USA; ^5^Centre for Chemistry and Biotechnology, School of Life and Environmental Sciences, Deakin University, Pigdons Road, Waurn Ponds, VIC 3216, Australia

## Abstract

The therapeutic use of natural herbs is an ancient human civilization act and the numbers of people have reliance on their pharmacological properties and preferred to use the natural herbs. People also use to consume these herbs as supplements to energize, bolster, and eventually enhance sexual ability. Polyherbal formulation (PHF) is one of these herbal amalgams that can be used to treat sexual dysfunction including erectile dysfunction, impotence, ejaculation dysfunction, and hypogonadism. The pilot study was aimed at evaluating the capacity of PHF in enhancing the spermatogenic potential of oligospermic patients. Thirty-six male patients with oligospermia were enrolled and randomized either to treatment (n = 23) with PHF (750 mg/d in three doses for 90 days) or to placebo (n = 13) in the same protocol. The preintervention semen analysis was compared with posttreatment semen analysis. Based on the postintervention semen analysis, patients were advised to undergo either* in vitro* fertilization (IVF) or intracytoplasmic sperm injection (ICSI) to assess their fertility status. After polyherbal treatment, there was a 256% increase in sperm concentration (9.59 ± 4.37 × 106/mL to 25.61 ± 8.6 × 10^6^/mL; P ≤0.001), 154% increase in semen volume (1.7 ± 0.14 mL to 4.32 ± 0.38 mL; P ≤0.001), and 215% increase in sperm motility (15.43 ± 2.40% to 48.65 ± 5.10%; P ≤ 0.001) on day 90 from baseline. Furthermore, a significant improvement and regulation were also observed in serum hormone levels with PHF treatment as compared to the placebo group. The present study demonstrated the evidence on synergistic spermatogenic effect of PHF as attributed in ayurveda for the treatment of oligospermia leading to infertility.

## 1. Introduction

Among human infertility, male infertility accounts for almost 50%. Among 40% to 50% of these infertile males, the etiologies remain unidentified [1–7]. Approximately 8% of these infertile men are looking forward to medical treatment to resolve infertility related issues [8]. Male infertility is caused by a wide range of etiologies. Numerous factors are defined with unequivocal and harmful effect on male reproduction function, i.e., (i) modern (sedentary) life style problems like obesity, smoking, caffeine, alcohol, and drug abuse, (ii) nutritional deficiencies including vitamin and mineral deficiencies [9] and oxidative stress [10], (iii) genetic causes related to male infertility like the cystic fibrosis transmembrane conductance regulator (CFTR) gene mutations leading to congenital absence of the vas deferens, Y-chromosome microdeletions in the azoospermia factor locus leading to spermatogenic impairment, and karyotype abnormalities, oncological diseases, (iv) hormonal issues encompass: hyperprolactinemia, hypothyroidism, congenial adrenal hyperplasia, hypogonadotropic hypogonadism, and panhypopituitarism, (v) physical problems likewise varicocele, damaged sperm ducts, torsion, Klinefelter's syndrome, retrograde ejaculation, psychological issues comprised of erectile dysfunction, premature ejaculation, and ejaculatory incompetence [11, 12]; hence, different approaches are required to resolve all these multifaceted dilemmas [13]. Oligospermia may be considered as a pathologic effect of the above-mentioned causes and consequently oligospermia represents a cause of reduced male fertility [1, 2].

Broad range of strategies had been carried out to overcome the male infertility complications. Human chorionic gonadotropin (hCG) [14], treatment of follicle-stimulating hormone (FSH) [15], and usage of assisted reproduction technology (ART) are of the state-of-the-art options for these infertility issues [16]. However, shortcomings of the above stated treatments cannot be overlooked, which may include acceptability, high expenditure, and threat of diverse complications during surgical procedures [17]. Approximately half of the couples undergoing consultation for male infertility terminated ART treatment, mainly due to the physical and emotional pain resulting from the treatment [16]. Additionally, there were some concerns for the offspring conceived by means of ART, including* in vitro *fertilization (IVF) and intracytoplasmic sperm injection (ICSI) via microsurgical epididymal sperm aspiration (MESA) or percutaneous epididymal sperm aspiration (PESA) showed a higher risk of birth defects [11, 18]. Furthermore, many people over the globe consumed locally grown plants/herbs as nutritional supplements and various therapeutic herbal formulas have been clinically evaluated and approved for fabrication of drugs in developed and developing countries to energize, bolster, and enhance sexual ability eventually to treat various sexual disorders [17, 19–21]. In the outlook of oligospermia, diverse indigenous herbs have been claimed to have sexual arousal effect or even useful for the management of low sperm count [22–27]. However, these herbs proved limited potential for improving the major assessed parameters for semen; in addition less literature has been cited for potential herbal remedy for oligospermia [28–36]. Therefore, there is an immediate need to fabricate a safe and cost-effective therapeutic drug from novel natural sources that devoid of all limitations stated above from modern medicines or/medical techniques and herbal remedies.

Since PHF due to their synergistic effects and multiple biological functions demonstrated the vast advantages over single herbal formulation, therefore, in the present investigation, a PHF was made, based on recommended ayurvedic practicing knowledge/literature citation, which is root of* Chlorophytum borivilianum*, seeds of* Hygrophila spinosa T. Anders*, seeds of* Mucuna pruriens*, seeds of* Mimosa pudica*, sap of* Acacia senegal*, root of* Astragalus membranaceus*, seed coat of* Plantago ovate*, sap of* Bombax ceiba*, and root of* Eurycoma longifolia *and nonherbal constitute called “rock candy”. All stated constituents were fabricated into a therapeutic PHF to determine the effects on men infertility. Although, there were several evidences on animal models regarding the sex-stimulating effect of above stated herbs on male fertility and sperm quality and other combination of herbs [28–38]. However no clinical trial has been performed yet to investigate the spermatogenic effect on human. For the reason, a proposed research trial was conducted to determine the synergistic spermatogenic potential of polyherbal formulation on reproductive function of oligospermic male patients.

## 2. Material and Methods 

*Ethics Statement.* This study was approved by the Institutional Review Board on Human Research of University of Lahore Teaching Hospital, Lahore, Pakistan, with reference number (IRB-UOL-FAHS/B00112A). The study was conducted according to the declaration of Helsinki principles. Written informed consent for evaluation and use of their clinical data for scientific purposes was obtained from each patient prior to recruitment.

### 2.1. Polyherbal Formulation

In the present investigation, PHF was made by combining the root of* Chlorophytum borivilianum*, seeds of* Hygrophila spinosa* T. Anders, seeds of* Mucuna pruriens*, seeds of* Mimosa pudica*, sap of* Acacia senegal*, root of* Astragalus membranaceus*, seed coat of* Plantago ovate*, sap of* Bombax ceiba*, root of* Eurycoma longifolia*, and rocky candy in a ratio of 1:1:1:1:1:1:1:1:1:8 respectively (Table 1), to determine the effect of the prepared formulation on oligospermic patients.

### 2.2. Preparation of Extract

Samples were thoroughly washed with deionized water to remove any contamination, ground to make crude powder with the help of liquid nitrogen followed by individual ethanol, methanol, acetone, and water extraction. Solvents are selected based on their polarities, from high to low (*i.e.,* water>methanol>ethanol>acetone) as different classes of bioactive compounds possessed different affinities towards the solvents of different polarities.

For the extraction, an aliquot of crude powder (5 g) was accurately weighed into four 250-mL flasks and shaken overnight (16–20 h) with 50 ml of ethanol, methanol, acetone, and water individually in ratio of 1:10 at room temperature. After the overnight shaking, all samples were centrifuged at 4000 rpm for 10 minutes, followed by the vacuum filtration of supernatant through Whatman paper No. 1 (x2). The residue was then resuspended in another 50 ml of ethanol methanol, acetone, and water, respectively. The process was repeated twice. The combined filtrate was transferred to a preweighted glass plate and the organic solvents were evaporated by placing at hot air oven at 40°C for 4 hours after which freeze-drying of filtrate was performed using an Alpha 1–4 LD plus freeze-drying unit (Labconco, Germany). After lyophilization, dry weight of sample was recorded and stored at −80°C for further usage.

### 2.3. Standardization of Polyherbal Formulation (PHF)

PHF was standardized as per WHO guidelines. Thin Layer Chromatography (TLC) was executed for three different batches, and the R_f_ value was determined for standardization parameters. Quantitative analysis of raw material was also carried out for organoleptic characteristics, physicochemical parameters, and toxicological parameters.

### 2.4. Sample Collection and Laboratory Methodology

Semen sample was collected at morning by patient masturbation into sterile plastic specimen cup in the hospital and all semen values were determined with compliance with World Health Organization (WHO) recommendations at the time of recruitment [39, 40]. Subjects were instructed to abstain from ejaculation for at least 72 hours prior to producing the semen samples. Samples were left to liquefy for 30 minutes but no longer than one hour prior to performing the semen analysis. To determine semen volume, samples were left to liquefy in an incubator at 37°C, 5% CO_2_ for 30 minutes before volume was measured, and sperm concentration and motility parameters were measured by means of computer assisted semen analyzer (CASA) (Hamilton-Thorne Version 10HTM-IVOS) using 2 Chamber Leja slides (Leja, The Netherlands). Setting parameters and the definition of measured sperm motion parameters for CASA were established by the manufacturer. A 5 *μ*l well-mixed, homogeneous sample was applied to each chamber of the Leja slide. Two known concentrations of Accu-bead® + (Hamilton-Thorne, Inc., Beverly, MA, USA) were used each day for quality control and to confirm accuracy of the CASA counting. The Leja slide was placed on the warm CASA stage and analyzed. A manual count of the same sample was also performed and discrepancies >10% required a repeat count. On manual count, sperm concentration was obtained by averaging the total number of sperm in both chambers on the Leja slide. At least 100 sperm cells were counted for motility assessment. Reference values from the World Health Organization (WHO) were used to assess sperm concentration and motility [39]. Testosterone, LH and FSH levels of blood serum were assessed using the Immulite automated chemiluminescence immunoassay analyzer (Immulite; Diagnostic Products Corp., Los Angeles, CA, USA) according to manufacturer's instructions, at University of Lahore Teaching Hospital. For IMMULITE 1000 system analytical sensitivity was 0.1 IU/L for both LH and FSH. The intra- and interassay CVs were 6.3 and 9.4% for testosterone, 4.0 and 7.1% for LH, and 4.2 and 8% for FSH.

### 2.5. Subjects

This double blind, two-armed, placebo-controlled, randomized, comparable group trial with 1: 1 arbitrary allocation was prospectively conducted at University of Lahore Teaching Hospital, Punjab, Pakistan, between September 2016 and December 2016. Out of ninety-one (91) patients, thirty-six (36) oligospermic patients were selected for the current clinical trial after predefined scrutiny. All participants in both groups were neither alcoholic nor user for any tobacco product. All oligospermic patients between the ages of 22 and 40 years were registered after acquiring the written approval from them; they also had a background of accustomed sexual intercourse over 12 months with a sexually normal female partner. All oligospermic patients exhibited the sperm count between the range of 0.0 to 15.0 million /mL. Subject with a total sperm count below the 0.0 million/mL or greater than 15 million/mL were not considered. Subject having cardiovascular diseases (CD), erectile dysfunction (ED), varicocele, testicular hypertrophy, uncontrolled diabetes mellitus (DM), obesity, precise hepatic or renal disorder, and cerebrovascular problems or with previous history of partly obstructive oligospermia, pelvic fractures prosthetic surgery on the penis, and cryptorchidism were also debarred from the current clinical trial. Oligospermic patients with known allergic reaction to any constitute of polyherbal extract were also not included.

### 2.6. Randomization and Treatments

The subjects for trial were randomized to PHF treated group (*n* = 23) and placebo group (*n* = 13). The oligospermic patients in PHF treated group were administered 3 capsules per day (containing 250 mg of high spectrum of PHF-extract) orally for a period of 3 months (90 days) on the other hand, capsules containing 250 mg of corresponding placebo were given in the same way to the placebo group.

The constituents used for formulation of polyherbal extract for the current trial had been extracted with an exclusive processing technology for developing a great phytopharmaceutical feature that amplified the action of PHF-extract manifolds, ultimately providing pan-therapeutic effects.

### 2.7. Trial Visit and Assessments

Throughout the treatment phase of 90 days (3 months), the subjects were mandatory to present themselves at the trial hub at specified intervals: Visit 01 on Day 60 and Visit 02 on Day 90 (3 months). Complete physical examination was conducted at baseline (Day 0), height and weights were measured by physician balance beam scale with height rod, while respiratory rate blood pressure and body temperature were assessed by Vital Signs Monitoring System (VSMS) and semen analysis was done at predefined time intervals likewise at Day 0, then after 60 days and 90 days. Semen analyses were performed according to World Health Organization 2010 criteria [39, 40]. Serum testosterone, luteinizing hormone (LH), and serum follicle-stimulating hormone (FSH) levels were evaluated on Day 0 (baseline) and then after Day 90. The principal (primary) usefulness result was the advancement in the major semen parameters and serum hormone level from baseline (Day 0) after 90 days of treatment.

### 2.8. Statistical Methods

In the current study, placebo group and PHF treated groups were evaluated for change in the semen parameters from the baseline (Day 0) by using one-way ANOVA (with treatment as a factor). The acquired outcome was taken as insignificant if the* P* value exceeded to 0.05.

## 3. Results

### 3.1. Composition of Polyherbal Formulation

Recently, polyherbal formulations (PHFs) are gaining importance all over the globe due to their dynamic medicinal and therapeutic claims and demonstrated vast advantages over single herbal formulations (SHFs), in the outlook of aforementioned claims; we have prepared a novel polyherbal formulation (PHF) to recover the male patients from oligospermia, and its composition is described in Table 1.

### 3.2. Standardization of Polyherbal Formulation (PHF)

TLC was run for the ethanolic extract in the solvent system ethanol/acetone/water at 17:2:1 ratio and plates were visualized directly after drying under UV at 254 nm and 366 nm in UV TLC viewer and then sprayed the TLC plates with a solution of 37% formaldehyde in conc. sulfuric acid (1:10) (for alkaloids), 1% ethanolic solution of aluminum chloride (for flavonoids), and p-anisaldehyde-sulfuric acid reagent (for phenols, sugars, steroids, and terpenoids) according to standard protocols. There were six spots evidently observed in all three batches and R_f_ values were calculated as 0.82, 0.77, 0.7, 0.67, 0.53, and 0.31 in each replicate. All other factors likewise organoleptic characteristics, physicochemical parameters, and toxicological parameters were also compliance with WHO recommendations and elaborated in Table 2.

### 3.3. Demographic Parameters and Participants Distribution

Ninety-one infertile males (n= 91) were taken into consideration for a proposed research study. Thirty-six (n= 36) were finally selected for clinical trial. This trial demonstrated the statistics of those 36 oligospermic patients that were double blind randomly distributed into two groups, i.e., the high-concentration PHF treated group (*n* = 23) and placebo group (*n* = 13). The two groups were comparable in terms of demographic parameters and demonstrated all vitals in optimum range (Table 3).

### 3.4. Semen Parameters

**Figure 1:**
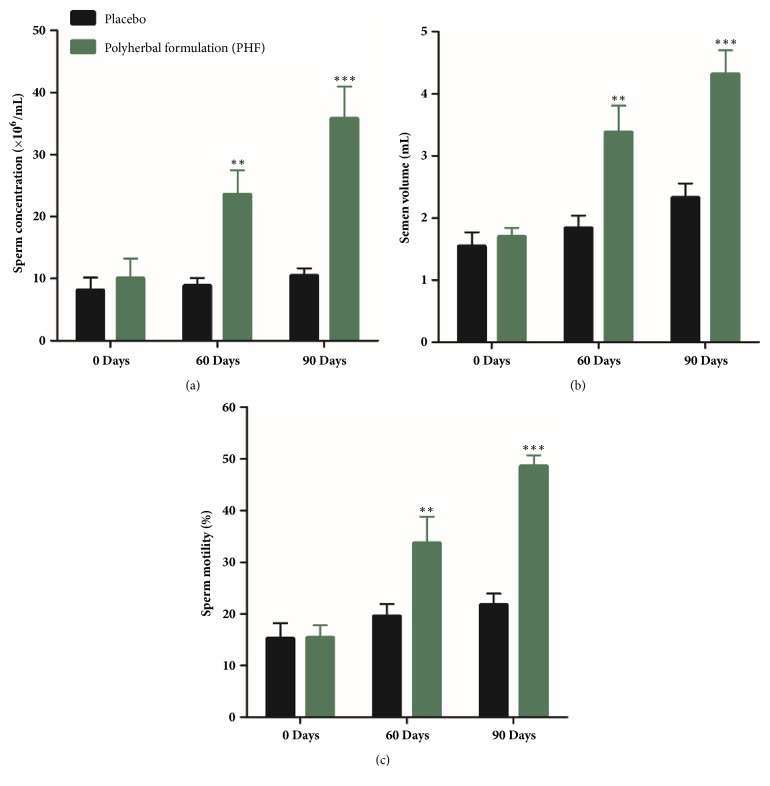
(a) Sperm concentration (×10^6^/mL), (b) semen volume (mL), and (c) sperm motility (%) in the full-spectrum PHF treated group and placebo group including oligospermic males. *∗∗P* ≤ 0.001 as compared to baseline values on Day 0 of the study duration of 3 months. Values are expressed as mean ± SD.

PHF treated group showed a highly significant (*P* ≤ 0.001) enhancement in sperm concentration after 90 days (3 months) of treatment as compared to the baseline value on Day 0 of the current trial (Figure 1(a)). The boost in sperm concentration was from 10.04 ± 3.21 × 10^6^/mL to 35.82 ± 5.11 × 10^6^/mL, corresponding to a percentage increase of 256%. A significant augmentation was also recorded in the semen volume (from 1.7 ± 0.14 mL to 4.32 ± 0.38 mL;* P* ≤0.001) (Figure 1(b)) and sperm motility (from 15.43 ± 2.40% to 48.65 ± 5.10%;* P* ≤ 0.001) (Figure 1(c)) on Day 90 (3 months) in contrast to the baseline value on Day 0. These corresponded to increases of 154% and 215%, respectively.

### 3.5. Serum Hormone Levels

**Figure 2:**
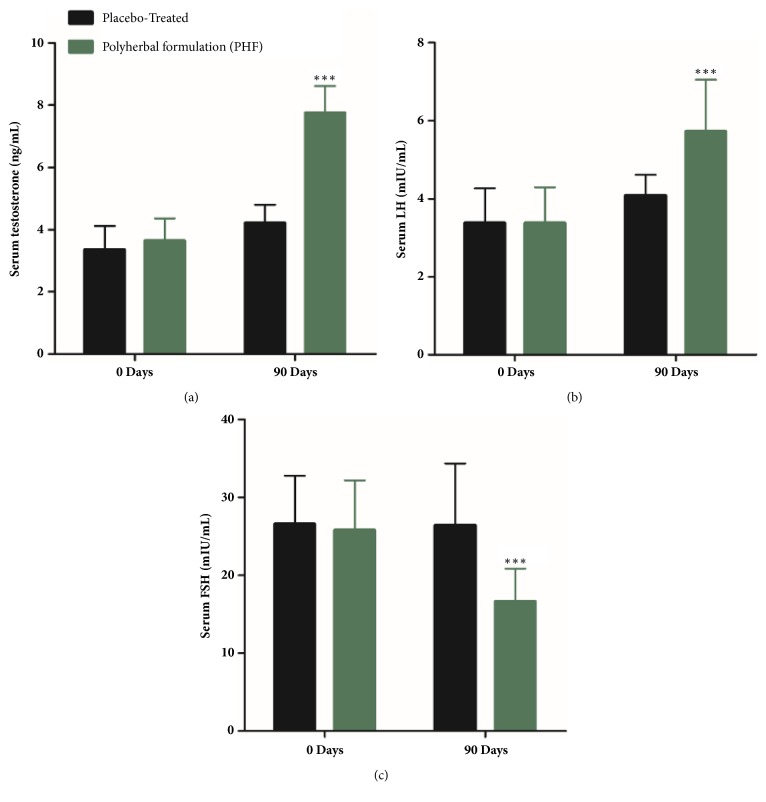
(a) Serum testosterone levels (ng/mL), (b) serum LH (mL IU/mL), and (c) serum FSH (mL IU/mL) in the full-spectrum PHF treated group and placebo group including oligospermic males. *∗∗P* ≤0.001 as compared to baseline values on Day 0 of the study duration of 3 months. Values are expressed as mean ± SD.

Moreover, serum hormones levels were also improved appreciably likewise testosterone level had been boosted up by 112% (from 3.65 ± 0.71 ng/mL to 7.76 ± 0.85 ng/mL;* P* ≤ 0.001) (Figure 2(a)) and LH by 69% (from 3.38 ± 0.91 mIU/mL to 5.73 ± 1.32 mIU/mL;* P* ≤0.05) (Figure 2(b)) while FSH level had been declined by 37% (from 26.45 ± 7.90 mIU/mL to 16.65 ± 4.21 mIU/mL;* P* ≤0.05) (Figure 2(c)) with subsequent treatment with PHF-extract, in contrast to the baseline (Day 0) values of these parameters.

## 4. Discussion

Infertility is defined as not being able to achieve pregnancy (conceive) after one year of isolated sex with the same partner. One year is lower reference limit (LRL) time for conceive decided by the World Health Organization [3, 41, 42]. Among infertility, male infertility accounts for about 50% of human infertility and considered to be less complicated than female infertility [3, 4, 43]. Several investigations had earlier confirmed that undermined semen production and sperm quality are amid the imperative causative factors for male infertility [5, 6]. In addition to different physical defects, oligospermia is the widespread cause of male infertility in more than 90% of the cases. Out of these, about 30 to 40% causes are unexplained, and in the rest of the cases critical illness, genetic abnormalities, malnutrition, pollution, side effects of some medicines, hormones, and chemicals play a key role in infertility [1, 2]. In the present investigation, we formulated a polyherbal formulation (PHF) based on previous therapeutic scientific endeavors for the critical oligospermic patients to recover their sexual potency. Among the key ingredients, we selected* Chlorophytum borivilianum* due to its aphrodisiac action, used to cure impotency and sterility and enhance male sexual potency ultimately for the treatment of certain forms of sexual inadequacies, such as premature ejaculation and oligospermia [28, 44]. Similar kinds of findings were also reported earlier, as discussed below in detail, by various investigators, and highlighted their positive outcomes on the oligospermia and different semen parameters using in vitro/in vivo model systems for* Hygrophila spinosa T. Anders *[29],* Mucuna pruriens *[30],* Mimosa pudica *[31],* Acacia Senegal *[32],* Astragalus membranaceus *[33],* Plantago ovate *[34],* Bombax ceiba *[35], and* Eurycoma longifolia *[36].

To the best of our acquaintance, this is the first demonstration to explore the synergistic efficacy of our prepared PHF on human clinical trial, which leads to the improvement in the major semen parameters of oligospermic males compared with placebo group. In this clinical study, we noticed highly significant improvements in major assessed parameters of semen such as sperm concentration, semen volume, and sperm motility in PHF treated group compared to placebo group. Serum hormones like testosterone, LH, and FSH were also markedly improved. Serum testosterone, LH, and FSH are the hormonal markers of androgenicity [45]. An elevated FSH level is generally designated to severe seminiferous epithelium damage [11] and it is inversely correlated with sperm concentration motility and morphology [46]. Among these biomarkers the serum testosterone and LH levels were increased up to 112% and 69%, respectively, and conversely FSH level was declined up to 37% in PHF treated group as compared to placebo group. The boost in serum testosterone and LH level and drop in FSH level may be predominantly attributed to herb* Mucuna pruriens* as it was documented that* Mucuna pruriens* increased epididymal alkaline phosphatase activity, protein level in epididymis, serum testosterone, LH, dopamine, adrenaline, and nor-adrenaline levels in infertile men and reduced the levels of FSH and PRL (Prolactin) [30, 47] and ultimately enhanced spermatogenesis due to the presence of high contents of alkaloids [48, 49]. Many authors also cited the antioxidant, antitumor, and anti-inflammatory impending of alkaloids [44]. The escalation in serum testosterone is also endorsed to another herb called* Eurycoma longifolia *which was previously reported for its ability to activate 17 *α*-hydroxylase/17, 20 lyase (CYP17) enzyme that would augment testosterone level [50]. In addition, another clinical study on human, also declared that* Eurycoma longifolia *supplementation increased the level of testosterone [51]. An increase in testosterone level has been associated with increase of sexual desire, penile tumescence, and rigidity, as well as the accessory muscles which help to strengthen and provide additional sexual activity [52–54]. Research with various animal and human models pointed out that there is a strong correlation between sexual behavior and brain neurotransmitters like dopamine, 5- hydroxytryptamine (5-HT), serotonin, and nitrergic neurons [55]. The motor switch of ejaculation in animals is also governed by serotonin and its receptors [56]. Testosterone may also expedite male sexual performance by aggregative dopamine discharge in the medial preoptic area and potentiating nitrergic neurotransmission in brain, which ensued in stimulation of hypothalamic-pituitary-gonadal axis [57, 58]. Hike in the testicular weight symbolizes the number as well as sperms motility [59]. Increased serum testosterone and LH levels and decreased FSH level after administration of PHF could thus be considered as one of the imparting factors accountable for the overall improved sexual potential against oligospermia in all PHF treated groups compared to placebo group.

The credit for a boost in endogenous testosterone levels and enhancing male sexual behavior goes to possible bioactive agents present in our novel formulation. These bioactive components may be steroids, antioxidants, saponins, peptides, organic acids, etc. and our future research will be comprised of characterizing and elucidating these novel bioactive compounds. The stated mechanisms of these agents include steroids by rising androgen production [60, 61], flavonoids by augmenting testosterone synthesis or by inhibiting its metabolic degradation [62, 63], alkaloids [52] by dilating the blood vessels in the sexual organs [64], and saponins by activating gonadal tissues and central nervous system via NO-dependent mechanism [65]. Thus, the improvements in sexual function established in the current study might be due to the existence of such compounds in our prepared PHF.

Sperm motility is also a very important factor for the proper fertilization and conceivability. Sperm motility was also significantly increased up to 215% as compared to placebo group in our study. This increase is mainly accredited by* Astragalus membranaceus *as at the dose of 10 mg/ml; the sperm motility in semen has increased up to 146.6 +/- 22.6% of control and for washed sperm increased up to 138.2 +/- 13.8% of control [33].

Nevertheless, sperm count and semen volume were also significantly increased up to 256% and 154%, respectively, in PHF treated as compared to placebo group. It may due to individual or synergistic activity of other PHF ingredients which had demonstrated previously proven spermatogenetic potential and aphrodisiac activity for alleviating oligospermia. Likewise, seeds of* Hygrophila spinosa* T. Anderson have spermatogenic effect which may be owing to the hormonal and neurohumoral changes, which play a vital role in the sexual behavior and fertility disorders [29].* Mimosa pudica* seeds extract possesses protective, therapeutic, and restorative capacity on the histoarchitecture of hypothalamic-pituitary-testicular axis components [31].* Acacia Senegal *enhanced the semen quality and could protect testis via improvement of antioxidant capacity; it may be useful to overcome the diabetic fertility complications [32].* Plantago ovate* seed used in considerable quantity in PHF ultimately facilitated recovering the oligospermia and subsidized the male infertility [34].* Bombax ceiba* root extract enhanced the spermatogenesis, sexual behavior, and erectile function [35].* Eurycoma longifolia* Jack plant may potentially be suitable for the management of oligospermia and male infertility [36, 66–68]. Our finding is also more significant compared with previously designed PHF which increased the sperm count maximum up to 39% to 40.5% in different animal models [37, 38].

It can be accomplished from above discussion that our currently designed PHF demonstrated more significant results compared with previous work on various animal and human models for individual component of current PHF or combination of two or more of other PHFs. In addition, aforementioned PHF also revealed diverse advantages over western medicine/medical techniques,* i.e*., higher expenditure associated with assisted reproductive technology (ART), ART affects patient's physical and emotional wellbeing may contribute psychological distress, caused birth defects, and proved less live birth rate [11, 17–36].

## 5. Conclusion

PHF could improve the quantity and quality of semen in a statistically significant manner in oligospermia male adults between the ages of 22 to 40 years, in comparison to the placebo, when used for 90 days, at 750 mg/d in three doses. PHF does also improve the serum testosterone; LH; and FSH level in a majority of PHF treated males, in comparison to the placebo. The results suggested that the prepared PHF may be a new auspicious novel therapeutic amalgamation, which can be used to improve the spermatogenic potential of many oligospermic infertile men. This spermatogenic property may be due to possible synergistic action of selected herbs' parts used in the preparation of PHF. However, further investigations are warranted to confirm and elucidate the effect of PHF on semen parameters.

## Figures and Tables

**Table 1:** Composition of polyherbal formulation.

**Scientific Name**	**Extraction material **	**Quantity (g/100 g of formulation)**	**Active Principles**
*Chlorophytum borivilianum*	Roots	5.88	Alkaloid, steroids
*Hygrophila spinosa* T. Anders	seeds	5.88	Terpenoids, phenolics
*Mucuna Pruriens*	Seeds	5.88	Alkaloids
*Mimosa pudica*	Seeds	5.88	Flavonoid, terpenoids, glycosides, alkaloids
*Acacia senegal*	Sap of tree	5.88	Polysaccharides
*Astragalus membranaceus*	Roots	5.88	flavonoids
* Plantago ovata*	Seed Coat	5.88	Psyllium mucilloid
*Bombax ceiba*	Sap of tree	5.88	Flavonoids, terpenoids
*Eurycoma longifolia*	Roots	5.88	Alkaloids, phenolics
Supersaturated Solution of Sugar and Water	-* *-* *-	47.05	-* *-* *-

**Table 2:** Standardization of polyherbal formulation (capsule).

**Name of Test**	**Observations**
**Organoleptic characteristics**	
**Description**	Grayish brown colored Powder filled in blue cap/red body, 0 sized capsule
Color	Grayish brown
Odor	Characteristics
Taste	Slightly Sweet
**Physicochemical Parameters **	
pH	7.6
Moisture Contents (%)	1.04
Average Weight (mg)	251
Weight Variations (Mean ± SEM)	249-253
Loss on drying (%)	1.2
Total ash (%)	6.54
Water soluble ash (%)	3.85
Acid-insoluble ash (%)	1.4
Ethanol Soluble Extractive value (%)	19.45
Methanol Extractive value (%)	15.02
Water Soluble Extractive value (%)	13.34
**Toxicological Parameters **	
Arsenic (ppm)	1.0 ppm
Lead (ppm)	0.5 ppm
Total microbial count NMT 1000 cfu/g	105 cfu/g
Yeasts and molds	Nil
Presence of *E. coli*	Absent
Presence of *Streptococcus*	Absent
Presence of* Salmonella*	Absent
Presence of *Pseudomonas*	Absent

**Table 3:** Demography and baseline data of the study subjects.

**Parameters**	**Placebo**	**PHF- Treated**
Age (yr)	28.53 ± 3.83	29.78 ± 4.75
Height (cm)	165.46 ± 9.05	165.47 ± 12.30
Weight (Kg)	72.46 ± 6.04	75.91 ± 7.10
Pulse (per min)	79.53 ± 6.56	81.39 ± 6.06
Respiratory Rate (per minute)	14.6 ± 1.45	14.34 ± 1.61
Systolic Blood Pressure (mm Hg)	123.66 ± 7.06	125.82 ± 4.09
Diastolic Blood Pressure (mm Hg)	84 ± 3.46	84.47 ± 2.59
Body Temperature (°F )	98.54 ± 0.30	98.60 ± 0.41

## Data Availability

The data used to support the findings of this study are available from the corresponding author upon request.

## Supplementary Materials

Supplementary Materials The graphical presentation of whole study has been provided in supplementary material.
