# Supplementary figures and images for: Evaluation of the Spermatogenic Activity of Polyherbal Formulation in Oligospermic Males
Source: Biomed Res Int. 2018 Jul 25;2018:2070895. doi: 10.1155/2018/2070895 (PMC6083514; doi:10.1155/2018/2070895)

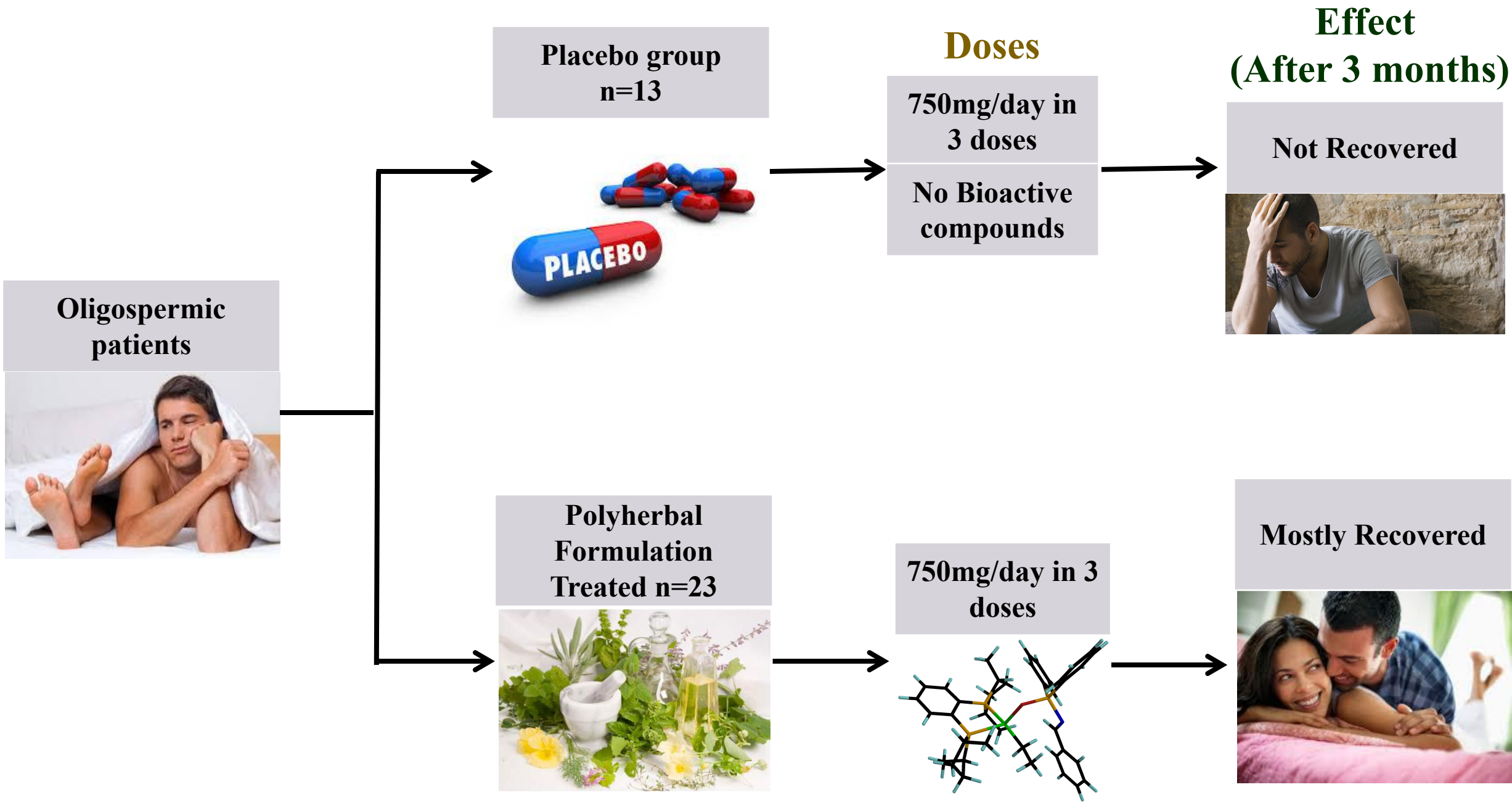

Supplement: Supplementary Materials — The graphical presentation of whole study has been provided in supplementary material. [file 2070895.f1.pdf]
